# Peptide–Drug Conjugates as Next-Generation Therapeutics: Exploring the Potential and Clinical Progress

**DOI:** 10.3390/bioengineering12050481

**Published:** 2025-04-30

**Authors:** Krishna Jadhav, Ashwin Abhang, Eknath B. Kole, Dipak Gadade, Apurva Dusane, Aditya Iyer, Ankur Sharma, Saroj Kumar Rout, Amol D. Gholap, Jitendra Naik, Rahul K. Verma, Satish Rojekar

**Affiliations:** 1 Institute of Nano Science and Technology (INST), Knowledge City, Sector-81, Sahibzada Ajit Singh Nagar, Mohali 140306, Punjab, India; krishnajadhavphd@gmail.com (K.J.); rahulverma@inst.ac.in (R.K.V.); 2 Department of Pharmaceutical Sciences, University of Connecticut School of Pharmacy, Storrs, CT 06269, USA; abhangashwin@gmail.com; 3 Department of Pharmaceutical Technology, University Institute of Chemical Technology, KBC North Maharashtra University, Jalgaon 425001, Maharashtra, India; koleeknath.111@gmail.com (E.B.K.); jitunaik@gmail.com (J.N.); 4 Department of Pharmaceutical Sciences, Delhi Skill and Entrepreneurship University, Dwarka Campus, Sector 9 Dwarka, New Delhi 110077, Delhi, India; deepscpn@gmail.com; 5 Department of Pharmaceutical Sciences and Experimental Therapeutics, College of Pharmacy, University of Iowa, Iowa City, IA 52242, USA; apurva-dusane@uiowa.edu; 6 Biopharmaceutics Department, Biocon Bristol-Myers Squibb R&D Center (BBRC), Bangalore 560099, Karnataka, India; adityaiyer251996@gmail.com; 7 Hyalo Technologies, Somerset, NJ 08873, USA; ankurshar2010@gmail.com; 8 Research and Development, LNK International Inc., New York, NY 11788, USA; sarojfnd@gmail.com; 9 Department of Pharmaceutics, St. John Institute of Pharmacy and Research, Palghar 401404, Maharashtra, India; amolgholap16@gmail.com; 10 Department of Pharmacological Sciences, Icahn School of Medicine at Mount Sinai, New York, NY 10029, USA

**Keywords:** peptide–drug conjugates, peptide, linkers, targeted therapeutics, stability, theranostic

## Abstract

Peptide–drug conjugates (PDCs) have emerged as a next-generation therapeutic platform, combining the target specificity of peptides with the pharmacological potency of small-molecule drugs. As an evolution beyond antibody–drug conjugates (ADCs), PDCs offer distinct advantages, including enhanced cellular permeability, improved drug selectivity, and versatile design flexibility. This review provides a comprehensive analysis of the fundamental components of PDCs, including homing peptide selection, linker engineering, and payload optimization, alongside strategies to address their inherent challenges, such as stability, bioactivity, and clinical translation barriers. Therapeutic applications of PDCs span oncology, infectious diseases, metabolic disorders, and emerging areas like COVID-19, with several conjugates advancing in clinical trials and achieving regulatory milestones. Innovations, including bicyclic peptides, supramolecular architectures, and novel linker technologies, are explored as promising avenues to enhance PDC design. Additionally, this review examines the clinical trajectory of PDCs, emphasizing their therapeutic potential and highlighting ongoing trials that exemplify their efficacy. By addressing limitations and leveraging emerging advancements, PDCs hold immense promise as targeted therapeutics capable of addressing complex disease states and driving progress in precision medicine.

## 1. Introduction

Peptide–drug conjugates (PDCs) have garnered significant attention in recent years as a promising strategy for targeted therapy. By combining the high specificity of peptides with the therapeutic potency of small drug molecules, PDCs offer a novel approach to enhancing drug efficacy while minimizing systemic toxicity [1]. The successful development of antibody–drug conjugates (ADCs) has demonstrated the potential of conjugate-based therapeutics for delivering drugs directly to sites of action. However, unlike antibodies, peptides are smaller, more versatile, and can penetrate tissues more effectively, making them attractive carriers for targeted drug delivery [2,3]. The growing interest in PDCs stems from their unique ability to address several limitations of conventional therapies. Small drug molecules often lack specificity, leading to off-target effects and a limited therapeutic index. On the other hand, peptide-based therapies, while selective, are frequently limited by their low stability and rapid degradation in systemic circulation [4,5,6]. PDCs bridge this gap by leveraging the best of both modalities—utilizing peptides for their specific targeting capabilities and small molecules for their potent therapeutic activity.

**Figure 1:**
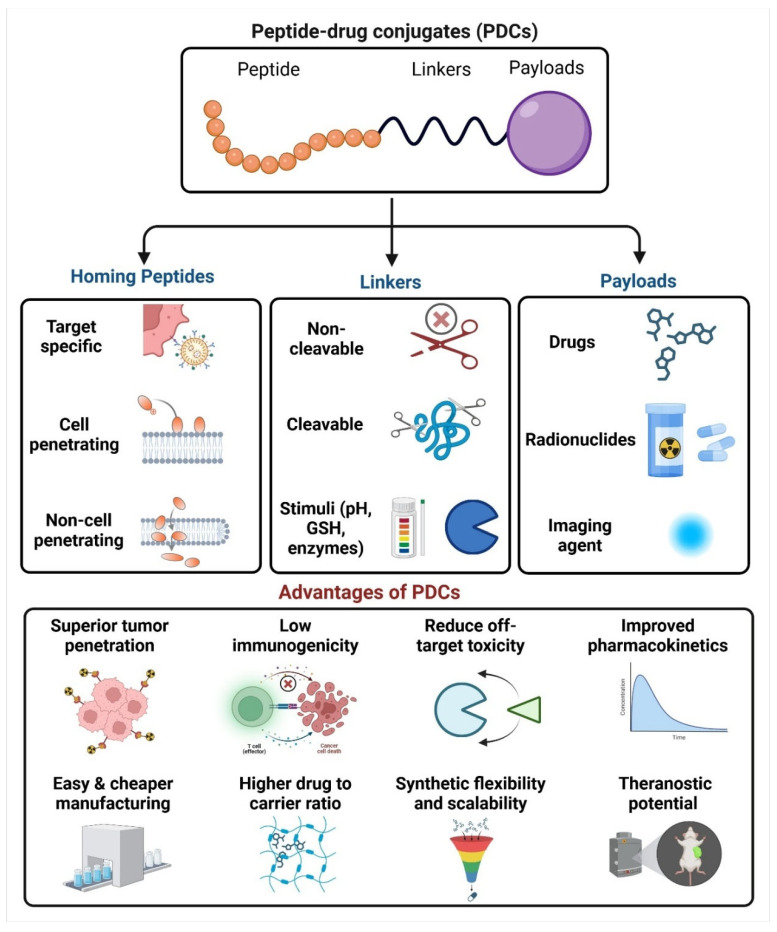
Schematic representation of a peptide–drug conjugate (PDC), comprising three key components: a homing peptide for targeted delivery, a cleavable or stable linker, and a therapeutic payload. PDCs offer several advantages over antibody–drug conjugates (ADCs), including smaller size for deeper tissue penetration, faster clearance reducing off-target toxicity, simpler and more cost-effective synthesis, and improved stability and versatility in targeting diverse receptors. Created with BioRender.com.

The design and development of PDCs involve several vital considerations, including selecting appropriate peptides and small molecules, choosing linker chemistry, and optimizing the drug-to-peptide ratio. Researchers can design PDCs that offer greater efficacy, enhanced selectivity, and improved safety profiles through precise engineering of their components (Figure 1) [7]. A critical aspect of PDC design involves the selection of appropriate homing peptides that can specifically bind to target receptors overexpressed on diseased or affected cells. These peptides are conjugated to small drug molecules through linker chemistry that are either cleavable or non-cleavable [8]. The choice of linker chemistry is crucial, as it dictates the stability of the conjugate in circulation and its ability to release the drug upon reaching at the target site [9]. Additionally, the choice of payload, such as cytotoxic agents, anti-inflammatory drugs, and imaging agents, allows the conjugate to be tailored for specific applications across various therapeutic areas [8]. The therapeutic scope of PDCs is broad, with applications extending beyond oncology to include the treatment of infectious diseases, neurological disorders, and inflammatory conditions. The ability of PDCs to address complex disease states, particularly those with established resistance mechanisms, has positioned them as a versatile tool in modern drug development [10]. By conjugating small-molecule drugs to peptides, it is possible to improve their pharmacokinetic properties. This can lead to increased drug exposure at the target site and prolonged therapeutic effects. As the field continues to evolve, several PDCs have progressed into clinical trials, showcasing promising results that highlight their potential to redefine targeted therapies [11,12,13].

This review will explore the core principles of PDC design, focusing on selecting peptide carriers, linker chemistry, and drug payloads. It will also examine strategies to optimize PDC efficacy, including enhancing cellular uptake, improving stability, and achieving tissue-specific targeting. Furthermore, the review will discuss the diverse applications of PDCs in treating cancer, infectious diseases, neurodegenerative disorders, and autoimmune conditions. Lastly, it will address the challenges and limitations of PDCs, such as safety, toxicity, and delivery barriers.

## 2. Fundamentals of Peptide-Based Drug Conjugates

### 2.1. Homing Peptides

Homing peptides are short, naturally occurring peptide sequences identified through biopanning techniques. These peptides can bind to specific receptors on the surface of cancer cells, angiogenic or remodelling blood vessels, or other cell types of interest, whether healthy or diseased [14,15]. Upon binding to these receptors, homing peptides, or nanoparticles decorated with them, can be internalized into cells through two primary methods: energy-dependent translocation and endocytic cellular uptake. Endocytosis mechanisms are common internalization pathways, including macropinocytosis, clathrin-dependent endocytosis, and caveolae-mediated endocytosis [16,17]. Additionally, a newly described internalization pathway, the C-end Rule (CendR) pathway, has been identified for tumor-homing peptides like CGKRK and CRGDKGPDC (iRGD) [17,18].

Homing peptides offer several advantages over antibodies as affinity-targeting ligands. Their small size facilitates better tissue and cell penetration and reduces immunogenicity. Furthermore, peptides typically interact with conserved and biologically significant binding pockets on target molecules, often resulting in functional activity [19,20]. To enhance stability and target binding, and introduce functional groups for site-specific conjugation, homing peptides can be engineered with non-natural modifications [3,21]. In some instances, proteolytic processing is essential for triggering peptide interactions with their targets. For example, the CendR peptides, which facilitate tumor cell penetration, require cleavage by a tumor-derived protease to expose their C-terminal arginine residue. Moreover, to extend their in vivo half-life, peptides can be conjugated to albumin-binding elements or polyethylene glycol or modified through amino acid substitutions and cyclic structures. In preclinical studies, peptide-based targeting has significantly improved payloads’ biodistribution and therapeutic efficacy. However, translating these results into clinical success remains a challenge, with FDA-approved peptide-targeted therapies still few [22,23,24].

Some homing peptides also function as cell-penetrating peptides (CPPs), which possess hydrophobicity, amphipathicity, and a net positive charge, facilitating their transport across cell membranes. When attached to cargo, such as therapeutic peptides or small molecules, these peptides guide the cargo to the desired tissue and enable its internalization. However, cationic CPPs can lack specificity, leading to non-selective uptake. To enhance tumor specificity, anionic CPPs are often incorporated in PDCs, and their targeting ability can be further improved with stimulus-responsive linkers [25,26,27]. Over the years, numerous peptides derived from the Arg-Gly-Asp (RGD) motif, along with integrin-specific ligands, have been meticulously engineered to enable the precise delivery of therapeutic agents and imaging probes. These peptides, including cRGDfV, cRGDfK, and RGD4C, target integrins such as αvβ3 and αvβ5, which play pivotal roles in angiogenesis. Preclinical studies have tested RGD-containing peptides in various cancer models, demonstrating their potential in designing drug delivery systems [27,28,29,30,31]. The Asn-Gly-Arg (NGR) homing motif, identified through peptide phage biopanning against α5β1 integrins, binds specifically to aminopeptidase N (CD13), which is overexpressed in tumor blood vessels and other pathological conditions. The NGR peptide has been utilized to deliver anticancer drugs and imaging agents to tumors, with significant therapeutic outcomes observed in mouse models of neuroblastoma, prostate, lung, and ovarian cancers [32,33,34,35]. Another homing peptide, Lyp-1 (CGNKRTRGC), binds to p32, a mitochondrial chaperone displayed on tumor endothelial cells and macrophages. Lyp-1 has been evaluated in preclinical studies for targeted delivery of paclitaxel (PTX)-albumin nanoparticles and doxorubicin (DOX)-loaded liposomes, demonstrating its potential for lymphatic tumor targeting [36,37,38]. The CREKA peptide, identified through phage display screening, binds to clotted plasma proteins in the tumor stroma and malignant blood vessels. It has shown enhanced antitumor and antimetastatic efficacy in models of metastatic breast cancer when used to target DOX-loaded liposomes [39,40]. Ultimately, the epidermal growth factor receptor (EGFR) family, comprising HER1, HER2, HER3, and HER4, is evidently overexpressed across a wide spectrum of solid malignancies. The EBP peptide, designed based on the structure of EGF, has shown improved anticancer efficacy and reduced systemic toxicity when conjugated with DOX in breast cancer xenograft models [41,42,43].

Several databases are available that compile information on PDCs. Among them, ConjuPepBD is a freely accessible and manually curated database that provides detailed annotations, including CAS numbers, biomedical applications, chemical conjugation types, and structural and physicochemical properties of PDCs [44]. In addition, the “PDCdb” knowledge base was developed, systematically compiling extensive information on PDCs, including biological activity data [45].

### 2.2. Linkers in PDC

Linkers serve as the crucial connection between drugs and peptides in PDCs, significantly influencing their circulation time and stability in vivo. An ideal linker should be stable during circulation to prevent premature drug release yet capable of releasing the drug rapidly and efficiently upon reaching the target tissue. Furthermore, the linker should not interfere with the peptide’s affinity for its receptor or the drug’s activity. The synthesis of linkers with peptides and drugs should be straightforward, maintaining stability throughout the process. Notably, the linker should not be overly hydrophobic, as this could lead to PDC aggregation, resulting in poor stability, reduced efficacy in vivo, and increased systemic toxicity and immune side effects. Linkers are generally classified based on their drug release mechanism and cleavage behavior into non-cleavable and cleavable linkers. Cleavable linkers, which include pH-sensitive, redox-sensitive, and enzyme-sensitive linkers, are specifically designed to break down under particular conditions within the tumor environment or target tissue, facilitating controlled drug release. Non-cleavable linkers, on the other hand, remain stable during circulation, providing advantages in plasma stability and reduced off-target toxicity [2,9,46].

#### 2.2.1. Non-Cleavable Linkers

Non-cleavable linkers, as their name suggests, are known for their stability and remain intact during circulation in the bloodstream. Their advantages over cleavable linkers include plasma stability, reduced off-target toxicity, a wider therapeutic window, and enhanced drug resistance. Non-cleavable linkers typically do not respond to external stimuli but release drugs through peptide metabolism, allowing them to maintain stability in the bloodstream until they reach their target sites [47,48]. Commonly used non-cleavable linkers for constructing PDCs include oxime bonds and thioethers [49]. To demonstrate the stability of thioether bonds under physiological conditions, Liang et al. engineered three distinct PDCs: RSSDOX, RSDOX, and RVCDOX. Each construct incorporated DOX as the cytotoxic payload and cyclic RGD (cRGD) as the targeting moiety, while employing different linker strategies, a redox-responsive cleavable disulfide bond (SS), a non-cleavable thioether linkage (S), and a valine–citrulline (VC) dipeptide linker cleavable by cathepsin B, respectively. From the drug release profiles, it is evident that the RSDOX conjugate, linked by a non-cleavable thioether bond, exhibited sustained and slow-release behavior. Notably, the drug release curve of RSDOX remained unchanged even after adding a small-molecule reducing agent (DTT), underscoring the stability of the thioether bond. Consequently, PDCs using such non-cleavable linkers maintain the integrity of the linker until reaching the target tissue, thereby preserving the drug’s cytotoxic efficacy. The selection between non-cleavable and cleavable linkers is dictated by the specific design objectives and strategic design of the therapeutic construct [9,50,51].

#### 2.2.2. Cleavable Linkers

Cleavable linkers are widely utilized in the construction of PDCs because they can be enzymatically or physiologically cleaved to release drugs at the targeted site. This key feature distinguishes them from non-cleavable linkers, offering the primary advantage of selective breakdown in the target tissues, which leads to controlled and rapid drug release. By ensuring that the drug is released specifically at the tumor site, cleavable linkers enable the achievement of therapeutic drug concentrations at the intended site of action while minimizing exposure to non-target tissues. This selective release mechanism exemplifies the concept of a “smart” drug delivery system designed to enhance therapeutic efficacy while reducing the toxic side effects associated with traditional treatments [2,50].

#### 2.2.3. pH-Sensitive Linkers

pH-sensitive linkers are designed to exploit the acidic conditions of the tumor microenvironment (pH 6.5–6.9) compared to normal blood pH (7.2–7.4), enabling controlled drug release specifically at the tumor site. These linkers remain stable in circulation but degrade rapidly in acidic conditions, ensuring that drugs are released predominantly within the tumor. Among the various pH-sensitive linkers, hydrazone bonds are the most extensively studied due to their sensitivity to acidity and effectiveness in achieving targeted drug delivery [2,52]. For instance, Saghaeidehkordi et al. developed an 18–4 peptide targeting breast cancer cells’ keratin 1 (K1) receptor. This peptide was conjugated to DOX via a pH-sensitive hydrazone bridge, creating a PDC. In their study on triple-negative breast cancer, mice treated with the PDC showed 1.4-fold higher DOX accumulation in tumors and 1.3–2.2-fold lower DOX levels in other organs. This resulted in significantly enhanced antitumor efficacy and reduced off-target toxicity compared to free DOX or saline treatments [9,50]. Despite the hydrazone bond’s popularity, its instability in blood circulation can lead to premature drug release, potentially diminishing therapeutic efficacy and causing unintended damage to non-target tissues [9]. An alternative pH-sensitive linker, acetal, has shown promising results due to its higher sensitivity to lower pH levels. Studies have demonstrated that the bond breakage rate of acetal increases ten-fold for every unit with a decrease in pH. Gillies et al. developed four conjugates using various acetal structures to link model drug molecules to PEO, assessing their hydrolysis kinetics through HPLC. The results indicated varied half-lives at pH 5.0, ranging from less than a minute to several days, with all conjugates showing slower hydrolysis at pH 7.4. This suggests that acetal bonds have significant potential in constructing pH-sensitive PDCs for more controlled and targeted drug release [53,54].

#### 2.2.4. Redox-Sensitive Linkers

Glutathione (GSH), a potent intracellular reducing agent, is pivotal in redox-sensitive drug delivery systems. With intracellular concentrations approximately 1000 times higher than extracellular levels, GSH is particularly abundant in tumor cells due to their hypoxic and abnormal microenvironment, which enhances reductase activity and elevates GSH levels. This unique characteristic of tumor cells makes GSH a critical trigger for cleaving specific chemical bonds used in drug conjugates. Notably, GSH’s antioxidant properties enable it to cleave bonds such as disulfide, thioesters, diselenides, and metal-thiol junctions, facilitating targeted drug release [9,55]. Among these linkers, disulfide bonds are widely employed in PDC construction due to their stability in systemic circulation and efficient cleavage in the reductive intracellular environment of tumors. Wu et al. leveraged this property to develop two PDCs, RGD-VC-CA and RGD-SS-CA, which incorporated a tumor-targeting peptide. While RGD-VC-CA used an enzyme-responsive dipeptide linker, RGD-SS-CA utilized a redox-sensitive cleavable disulfide linker. In vitro studies revealed that RGD-SS-CA outperformed RGD-VC-CA in drug release and cytotoxicity, demonstrating the superior efficacy of redox-sensitive linkers for tumor-specific drug delivery. Furthermore, in vivo experiments on tumor-bearing mice showed significant tumor growth inhibition with RGD-SS-CA administered intravenously, highlighting its potential as an effective and targeted therapeutic strategy [56].

#### 2.2.5. Enzyme-Sensitive Linkers

Enzyme-sensitive linkers play a crucial role in PDCs by enabling targeted drug release in response to specific enzymatic activity. These linkers often rely on chemical bonds or peptide sequences that degrade selectively under enzymatic action. Chemical bonds such as ester, amide, and carbamate are widely employed in PDC designs due to the abundance of esterases and amidases in tumor cell endosomes and lysosomes, which facilitate drug release. Among these, ester and amide bonds are particularly popular for constructing tumor-targeted PDCs [9,57]. Bohme et al. demonstrated this approach by linking neuropeptide Y (NPY) analogs to methotrexate using amide bonds, where the antitumor activity was proportional to the quantity of methotrexate conjugated through these bonds [58].

Similarly, carbamate linkers are valued for their stability and ability to undergo trace-free hydrolysis, producing carbon dioxide, amines, and alcohols during drug release, ensuring efficient delivery without residual fragments. In addition to chemical linkers, enzyme-sensitive peptide linkers provide another strategy for selective drug release. These linkers degrade specifically under the action of tumor-associated enzymes, such as proteases, to release the active drug. For example, tumor cells enriched with enzymes like cathepsin B in their endosomes and lysosomes effectively hydrolyze peptide bonds. Unlike enzyme-responsive peptides, which modify PDC structure upon enzyme interaction, enzyme-sensitive peptide linkers break down completely to liberate the drug, ensuring precise and efficient delivery at the tumor site [59].

### 2.3. Payloads of PDC

The payloads of PCDs typically consist of cytotoxic or therapeutic agents designed to exert targeted effects. Despite their potential, these drugs often face challenges, such as low water solubility, poor selectivity, short half-life, and instability, which limit their clinical applicability. For successful delivery through PDCs, the drugs must have appropriate attachment sites and remain inactive in their conjugated form, activating only upon release at the target site. This ensures a well-defined mechanism of action and potent pharmacological activity. Conjugation with peptides addresses these limitations by improving solubility, enhancing selectivity, extending circulation time, optimizing bioavailability, and reducing off-target side effects and toxicity. Cytotoxic payloads in PDCs are characterized by low IC50 values, typically in the nanomolar range, and include examples like PTX, 5-fluorouracil, DOX, and daunorubicin [37,60]. Additionally, radionuclides can serve as either therapeutic or diagnostic payloads. For example, [^68^Ga]Ga-dotatate is commonly used for imaging, while [^177^Lu]Lu-dotatate, an FDA-approved therapeutic agent, is used for targeted radionuclide therapy. In therapy, PDCs also function as imaging agents. A notable example is the FDA-approved radionuclide-containing PDC, In-DTPA-Octreotide (Octreoscan), used for diagnosing neuroendocrine tumors. However, Octreoscan’s therapeutic potential is limited, as it primarily aids in diagnosis rather than tumor regression [61,62,63].

## 3. Targeting, Binding, Internalization, and Drug Release at Target Sites

Targeting, binding, and internalization of a PDC into target cells, followed by the release of the drug payload at the target site, are processes influenced by multiple factors, including the type of linker and homing peptide used. The mechanism of action varies depending on these elements. Cleavable linkers play a crucial role, as they can be activated by specific stimuli such as pH changes or the presence of enzymes, dictating the site and method of drug release. In one scenario, reminiscent of ADCs, the PDC undergoes internalization, followed by intracellular cleavage to release the drug payload. Alternatively, cleavage can occur extracellularly, subsequently enabling the free drug to enter target cells [36,54]. Homing peptides are another critical determinant of the PDC’s mechanism of action. These peptides can be categorized into cell-penetrating and non-cell-penetrating types. Non-cell-penetrating homing peptides typically bind to overexpressed receptors on tumor cells, initiating receptor-mediated endocytosis. Once internalized, the PDC dissociates from the receptor in the early sorting endosome. It is then transported to the late endosome and eventually to the lysosome, where the acidic environment or specific lysosomal enzymes cleave the PDC, releasing the cytotoxic drug [14,15]. Other mechanisms have also been reported. For instance, You et al. described a PDC designed for targeting metastatic breast cancer, where matrix metalloproteinase-2 (MMP-2) cleaves the PDC in the tumor microenvironment before internalization. Following cleavage, the released drug—DOX—diffuses across the tumor cell membrane to exert its therapeutic effect [64]. Therefore, selecting the linker, homing peptide, and target tissue, along with considering the stimuli present in the microenvironment, is critical in designing a PDC to ensure the desired mechanism of action is achieved.

## 4. Applications of Peptide–Drug Conjugates (PDCs)

### 4.1. Cancer Therapy

Current chemotherapy using anti-cancer agents suffers from significant drawbacks such as undesired side effects, lack of selectivity, and non-adherence to dosing regimens. ACPs offer a promising alternative to overcome these limitations, providing an effective therapeutic approach against tumors. These low-molecular-weight bioactive peptides, typically composed of 10–50 amino acids, can be derived from natural sources or synthesized chemically, targeting various anti-tumor mechanisms [65,66,67]. These mechanisms include damaging genetic material [68], disrupting membrane integrity [69], inducing programmed cell death [70], and arresting angiogenesis [71].

#### 4.1.1. Cell Targeting Peptide–Drug Conjugates

**Figure 2:**
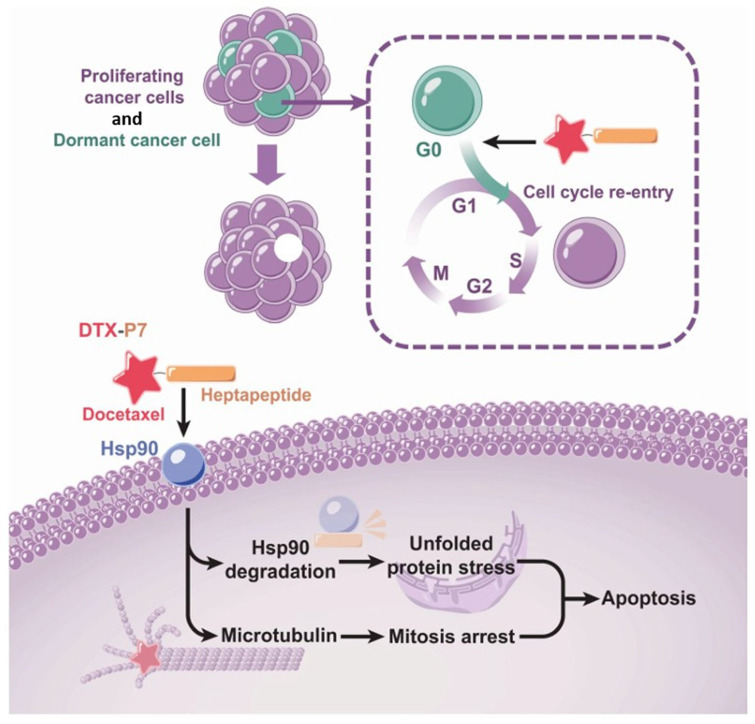
DTX-P7 suppresses tumor growth and triggers apoptosis in tumor cells by selectively accumulating in tumor tissues, promoting Hsp90 degradation, and activating the unfolded protein response. Image reproduced with permission from Jiang et al. [75].

To effectively target specific biochemical features of cancer cells and differentiate them from healthy normal cells, a thorough understanding of the pathology and physiological changes surrounding cancer cells is essential. Cancer cells exhibit distinct characteristics that can be exploited for therapeutic interventions. These include the dysregulation of translational machinery [72], alterations in epigenetic regulatory mechanisms [73], and the overproduction of enzymes crucial for their survival and evasion of the body’s natural defense mechanisms against uncontrolled proliferation [74]. Additionally, cancer cells often overexpress specific receptors unique to particular types of cancers, aiding in assimilating essential nutrients and chemicals. Another hallmark is the shift in pH surrounding cancer cells toward a more acidic environment. This acidic microenvironment plays a role in attenuating the release of cytokines, further contributing to immune evasion. These distinct features present valuable opportunities for designing PDCs that selectively target cancer cells while sparing healthy tissues. A significant area of research focuses on overexpressed receptors, which serve as promising targets. These receptors are commonly exploited through cell-targeting peptides (CTPs), which enhance specificity in cancer therapy. The interaction between the attached peptide and its target receptor relies heavily on the precise amino acid sequence of the peptide, which ensures recognition and binding to the receptor. By integrating knowledge of these biochemical and physiological alterations, PDCs can be optimized to selectively target cancer cells, minimize off-target effects, and improve therapeutic outcomes. Jiang et al. designed a novel PDC (DTX-P7) was developed by conjugating docetaxel (DTX) with a heptapeptide (P7), which selectively binds to cell surface Hsp90. The anti-tumor efficacy of DTX-P7 was evaluated in non-small cell lung cancer (NSCLC), demonstrating enhanced tumor suppression compared to free DTX, along with preferential tumor accumulation and extended circulation time. Mechanistically, DTX-P7 was found to induce unfolded protein response (UPR), ultimately triggering apoptosis. Notably, it also stimulated the cell cycle reentry of slow-proliferating cancer stem-like cells (CSLCs), leading to their subsequent elimination, following a “proliferate-to-kill” strategy (Figure 2) [75].

#### 4.1.2. Integrins

Integrins are a family of transmembrane receptors that play a critical role in cellular adhesion, signaling, and cancer progression. Their overexpression in various cancers makes them attractive PDC targets. A significant advancement in targeting integrins was made by Chen et al. [76], who first synthesized RGD–PTX conjugates. These conjugates utilized cyclic dimeric RGD linked via an ester bond hydrolyzed intracellularly by lysosomal enzymes to release PTX. While the RGD–PTX conjugates demonstrated enhanced in vitro cell penetration and retention compared to PTX alone, their high lipophilicity caused low solubility, leading to poor in vivo bioavailability and no significant tumor volume reduction. To further evaluate the biodistribution of these conjugates in MDA-MB-435 breast cancer cells, iodine was attached as a radiolabeled ligand. Rapid blood clearance was observed for RGD and RGD–PTX conjugates, with higher accumulation in tumor cells [76]. This highlighted the potential for targeting but also underscored the need to optimize drug delivery systems. Building on this, researchers synthesized DOX-RGD PDCs by attaching a prodrug of DOX to the RGD peptide. These conjugates showed improved affinity for the αvβ3 integrin receptor in vitro. Their anti-tumor potency in MDA-MB-435 cells proved superior to DOX alone, further emphasizing the role of RGD peptides in enhancing receptor-specific drug delivery [77].

Beyond RGD peptides, Cox et al. introduced another peptide carrier molecule, knottins, which displayed selective binding to integrins, particularly αvβ3-, αvβ5-, and αvβ1-conjugating Knotting with gemcitabine. The synthesized Knottin-Val-Ala-PAB–Gemcitabine conjugate exhibited superior tumor growth inhibition across various cell lines, showcasing the versatility of integrin-targeting peptides in cancer therapy [55]. Further advancements included the development of a PDC targeting the αvβ6 integrin receptor, overexpressed in many cancers, to enhance tumor-specific drug delivery and minimize systemic toxicity. This PDC employed monomethyl auristatin E (MMAE) as the cytotoxic payload linked to an αvβ6-binding peptide. The conjugate exhibited high stability, selective tumor cell internalization, and effective cytotoxicity. Tumor-specific accumulation was visualized using PET imaging, enabling simultaneous therapeutic and diagnostic capabilities. In vivo studies demonstrated prolonged survival in mice bearing αvβ6-positive tumors, highlighting the potential of this dual-function PDC for targeted cancer therapy and real-time treatment monitoring [78].

#### 4.1.3. Somatostatin Receptors

Somatostatin receptors (SSTRs), particularly SSTR2, are overexpressed in various cancers, making them valuable targets for PDCs. Redko et al. developed a novel somatostatin (SST) peptide analog, 3207-86, with high selectivity for SSTR2. This analog was conjugated with five chemotherapeutic agents, each acting through distinct anti-tumor mechanisms. The conjugates, linked via a disulfide-bridged cyclic conformation of 3207-86, demonstrated exceptional biological stability and enhanced anti-tumor efficacy in SSTR2-overexpressing HCT116, H1299, and TRAMP C2 cell lines [79]. Expanding on this, Whalen et al. synthesized PEN-221, a PDC that combines the somatostatin analog Tyr-octreotate with the cytotoxic agent DM1, using a disulfide linker at the peptide’s C-terminus [80]. PEN-221 exhibited significant efficacy even at a low dose (one-sixth of the maximum tolerated dose) and displayed dose-dependent activity. Both in vitro and in vivo studies demonstrated the internalization of PEN-221 in SSTR2-positive small cell lung cancer (SCLC) cell lines, affirming its potential as a targeted therapy [81]. Another notable SST analog, Octreotide, was synthesized by Bauer et al. This analog, comprising the sequence D-Phe-Cys-Phe-D-Trp-Lys-Thr-Cys-Thr(ol), demonstrated the highest affinity for the SSTR2 receptor. When conjugated with 111Indium, Octreotide became the first USFDA-approved PDC diagnostic agent, marketed as Octreoscan, for tumor imaging [82]. Building on Octreotide’s potential, Lelle et al. prepared an Octreotide–DOX conjugate. This conjugate showed specific binding to SSTR2 and greater efficacy in reducing tumor growth in MCF-7 breast cancer cell lines. Moreover, it effectively suppressed the secretion of ACTH in vitro from AtT-20 pituitary tumor cell lines, showcasing its dual therapeutic potential [83]. In another study, Camptothecin (CPT), a potent chemotherapeutic agent, was conjugated with the SST analog JF-07-069. This PDC exhibited remarkable selectivity and anti-tumor efficacy in SSTR2-overexpressing cell lines, including IMR32, CFPAC-1, MOLT-4, and PC-3. Notably, the PDC achieved more than an 80% tumor volume reduction across all tested cell lines, highlighting its therapeutic promise [84].

#### 4.1.4. Epidermal Growth Factor Receptor (EGFR)

The EGFR is a transmembrane protein that is abundantly overexpressed in a variety of malignancies, making it an essential target for therapeutic intervention. Panosa et al. developed a modified epidermal growth factor termed EGFt, which binds selectively to EGFR and internalizes, reaching the nucleus. EGFt facilitates drug delivery and inhibits the binding of native ligands to EGFR on cancer cells, thereby impeding cell proliferation and differentiation [85]. To improve targeting specificity, Li et al. [86] synthesized a novel EGFR analog known as the GE11 peptide (YHWYGYTPQNVI). GE11 exhibited high selectivity and effective internalization in EGFR-overexpressing cancer cell lines. The study explored gene delivery using a GE11-conjugated polyethylenimine vector, which demonstrated successful uptake in EGFR-positive cells and tumor xenografts, highlighting its potential in targeted gene therapy [86]. Ahsan et al. [87] further advanced EGFR-targeted therapy by synthesizing disruptin, a peptide comprising the SPDNPHVC segment of EGFR. Disruptin displayed selective internalization into cancer cells while sparing normal cells, addressing a critical limitation of earlier therapies. When administered intraperitoneally (IP) in nude mice bearing human EGFR tumor xenografts, disruptin promoted EGFR degradation without causing damage to host cells. Unlike traditional EGFR inhibitors such as geldanamycin or cisplatin, disruptin was shown to lack the associated toxicity, further reinforcing its therapeutic potential. These advancements in EGFR-targeting peptides highlight their capability to inhibit tumor progression while minimizing off-target effects, paving the way for safer and more effective [87].

#### 4.1.5. Bombesin Receptor Family

The bombesin receptor family, consisting of neuromedin B receptor (NMB/BB1), gastrin-releasing peptide receptor (GRPR/BB2), and BB3 receptor, plays an essential role in mammalian peripheral and central nervous tissues. GRPR is notably overexpressed in several cancers, including small-cell lung cancer (SCLC), breast cancer, exocrine tumors, and some glioblastomas, with expression levels exceeding 70% in SCLC. GRPR activation leads to the transactivation of EGFR, promoting cell proliferation and differentiation [88]. To explore the therapeutic potential of bombesin receptor targeting, Cescato et al. investigated two bombesin analogs, Demobesin 1 and Demobesin 4, for their affinity and functional activity towards GRPR. While Demobesin 1 acted as a potent antagonist, reversing the stimulatory effects of gastrin-releasing peptide (GRP) substrates, Demobesin 4 demonstrated higher internalization in HEK293 and PC3 cell lines, showcasing its potential as a tumor-targeting agent [89]. In another study, Jacopo et al. synthesized various bombesin analogs, including the novel peptide, [D-Phe^6^, β-Ala^11^, Sta^13^, Nle^14^] BBN_(6–14)_, and evaluated their drug delivery potential using daunorubicin as a model drug. These conjugates exhibited enhanced receptor binding and efficient cell internalization, demonstrating their safety and efficacy in MDA-MB-231, PC3, and MDA-MB-453 cell lines [90]. Further advancing bombesin receptor-targeted therapies, Terry et al. developed bombesin peptide analogs conjugated with marine toxins such as Hemiasterlin and Dolastatin. Using the peptide DPhe-Gln-Trp-Ala-Val-βAla-His-Phe-Nle-NH2 (BA1) as the carrier, these conjugates were tested in NCI-H1299 lung cancer cell lines. Both conjugates were effectively internalized; however, only Hemiasterlin conjugates inhibited cancer cell proliferation, establishing their cytotoxic potential against GRPR/BB2-expressing lung cancer cells [91].

#### 4.1.6. PDCs Acting on Immune Regulation (Immune Checkpoint Blockade)

The human body is equipped with a sophisticated immune system that employs T-cells and natural killer (NK) cells to combat foreign invaders and internal abnormalities such as cancer cells [92]. However, tumors evolve mechanisms to evade immune detection and suppression. These include reducing tumor-associated antigens, recruiting regulatory T-cells (Tregs), and promoting tumor-associated macrophages (TAMs), all of which dampen the immune response [93]. Activating immune cells within the tumor microenvironment represents a promising strategy to destroy cancer cells and prevent relapse. Tumor cells exploit immune checkpoints to suppress T-cell activity, notably through receptors such as programmed cell death protein 1 (PD-1) and cytotoxic T lymphocyte antigen-4 (CTLA-4) [94,95]. Overcoming this suppression using PDCs designed to target these pathways has emerged as a novel approach. Wang et al. synthesized a PD-L1-targeting peptide (PPA1) conjugated with DOX for colon cancer treatment [96]. This PDC utilized a pH-sensitive linker to release DOX selectively in the acidic tumor microenvironment, sparing normal cells. In a CT26 colon cancer xenograft mouse model, PPA1-DOX demonstrated superior safety, showing no significant reduction in body weight compared to DOX alone. Fluorescent labelling of the PDC confirmed high tumor specificity and accumulation. Immunohistochemical analysis revealed increased infiltration of CD4+ and CD8+ T-cells in the tumor microenvironment, leading to significant tumor size reduction. Pang et al. developed a poly(lactic-co-glycolic acid) (PLGA) nanoparticle formulation of M2pep peptide conjugated with PLX3397, a CSF-1 inhibitor, to target TAMs [97]. Moon et al. took an innovative approach by creating a PD-L1-targeting peptide (CVRARTR) conjugated with an immunogenic cell death (ICD) inducer, DOX, using a cathepsin B-specific cleavable FRRG peptide linker. This conjugate formed nanoparticles (Nano-IDCs) through interactions such as π-π stacking and hydrogen bonding. These Nano-IDCs exhibited high tumor accumulation via the enhanced permeability and retention (EPR) effect and PD-L1-mediated active targeting. Once internalized by receptor-mediated endocytosis, DOX was released in response to cathepsin B overexpression, inducing significant ICD and enhancing the expression of damage-associated molecular patterns (DAMPs). This increased tumor-infiltrating lymphocytes (TILs) in the tumor tissue. The Nano-IDCs also disrupted the PD-1/PD-L1 axis, amplifying pre-existing anti-tumor immunity while sparing normal and immune cells with low cathepsin B expression. These studies highlight the immense potential of PDCs in immune checkpoint blockade. By selectively targeting tumor microenvironments and activating immune responses, these strategies offer safe and effective combinational immunotherapy approaches to combat cancer [98].

#### 4.1.7. Other Advantages of PDC in Delivering Chemotherapeutic Agents

Most current anti-cancer agents belong to the Biopharmaceutics Classification System (BCS) Class II or IV, characterized by poor solubility and low permeability [99]. These physicochemical limitations present significant challenges for effective drug delivery, mainly through the oral route, which is the most common and patient-friendly method of drug administration. To be absorbed efficiently from the gastrointestinal (GI) tract, therapeutic agents must exhibit sufficient solubility and an optimal log P value to traverse lipid bilayers easily [100]. Therefore, understanding key physicochemical properties, such as solubility, log P, pKa, polymorphic forms, and stability in physiological conditions, is critical for drug development. An emerging strategy to overcome these limitations involves the use of peptide linkers. These linkers improve the solubility and stability of chemotherapeutic agents and enhance their selectivity and anti-tumor efficacy by facilitating targeted delivery [101]. One notable example is CPT, which suffers from poor solubility and lacks specificity for neoplastic cells [102]. Cui et al. addressed this by conjugating CPT with the tau-derived peptide linker VIQVIC. This conjugate self-assembles in aqueous environments to form nanotubes, enabling sustained in situ drug release [103]. Additionally, they developed an amphiphilic prodrug (DiCPT-iRGD) by incorporating the RGD peptide. The hydrophobic DiCPT resides on the inner surface of nanostructures, significantly enhancing solubility. The cytotoxicity assays showed that DiCPT-iRGD reduced the IC50 values of CPT by half in GL-261 brain cancer cell lines. This improvement is attributed to targeted internalization via neuropilin-1 binding, which enhances penetration into tumor cells [104]. Similarly, Gao et al. utilized a peptide-based strategy to enhance the properties of PTX. They conjugated PTX covalently with Nap-FFKYP (1-P) through a succinate linker, forming hydrogels for anti-cancer therapy. The hydrogel nanostructures extend the drug’s circulation time in the body, improving anti-tumor activity. The enzyme CES, overexpressed in HepG2 cells, cleaves the succinate bond, releasing PTX within the cells. Consequently, 1-Paclitaxel-P demonstrates better anti-tumor efficacy compared to PTX alone [105]. Further advancements include conjugating DOX with the hexapeptide KGFRWR. This conjugate self-assembles into nanofibers, facilitating sustained drug release and increasing circulation time. The DOX-KGFRWR nanofibers show enhanced anti-tumor efficacy compared to free DOX, highlighting the potential of peptide-based delivery systems for improving cancer treatment outcomes. These studies underscore the transformative potential of peptide conjugation in addressing the limitations of existing anti-cancer agents. By improving solubility, stability, and targeted delivery, peptide-based strategies pave the way for more effective and patient-friendly cancer therapies [106].

### 4.2. Antimicrobial Peptide–Drug Conjugate

PDCs have emerged as a powerful strategy for combating antimicrobial resistance by combining the targeting capabilities of peptides with the potency of antimicrobial agents. These conjugates enhance drug selectivity, stability, and efficacy while reducing systemic toxicity. Several PDCs have been developed to target drug-resistant bacterial infections, demonstrating significant therapeutic potential (Table 1). Rodriguez et al. developed a PDC by conjugating levofloxacin with Pep-4 at the N-terminus and lysine side chains. The resulting conjugates exhibited enhanced antibacterial activity compared to the unconjugated antimicrobial peptide (AMP). Additionally, the conjugates retained higher antibacterial efficacy in high-salt environments, a condition under which natural AMPs typically lose their activity [107]. Desgranges et al. designed β-lactamase-cleavable conjugates by linking cephalosporin with Bac8c to specifically target drug-resistant bacteria. Since β-lactamase, an enzyme produced by drug-resistant bacteria, degrades the β-lactam ring of antibiotics, cephalosporin was utilized as a masking agent for AMP activity. Upon cleavage of the β-lactam ring, the conjugate exhibited potent antibacterial effects [108]. In a study by Li et al., precursors of cephalosporins (7-aminocephalosporanic acid and 7-aminodesacetoxycephalosporanic acid) were conjugated to MSI-78, CA(1–7)M(2–9), and des-Chex1-Arg20 using a glycolic acid linker. This conjugation strategy enhanced antibacterial potency while reducing cytotoxicity in mammalian cells, demonstrating the potential of β-lactam antibiotic precursors in improving AMP effectiveness [109].

The choice of linkers plays a crucial role in reducing steric hindrance and enhancing molecular interactions. Ghaffar et al. investigated the effect of glycine and glycolic acid linkers on Indolicidin-TAT-Levofloxacin conjugates, reporting that Levofloxacin–Indolicidin conjugates with glycolic acid linkers exhibited superior antibacterial activity compared to those with glycine linkers [110]. Similarly, Umstatter et al. employed various PEGylated and long-chain linkers such as SMCC (SM(PEG)12), KMUS, and AMAS to conjugate polycationic peptides with vancomycin. The study found that Van3 conjugates with PEG linkers demonstrated enhanced antibacterial efficacy compared to other linkers, highlighting the importance of linker selection in optimizing antimicrobial activity [111]. Brezden et al. developed a cleavable AMP–antibiotic conjugate, linking kanamycin with P14LRR (Fl-PRPRPL-4) using a disulfide bond-dependent linker. Once internalized into the bacterial cell, the disulfide bond was cleaved, releasing two separate active components. The conjugate exhibited significantly enhanced antibacterial activity, showing a 128-fold reduction in the minimum inhibitory concentration (MIC) against kanamycin-resistant strains [112,113]. Similarly, HLopt2 was conjugated with fluconazole, levofloxacin, and ciprofloxacin using a disulfide-containing linker, resulting in a 4-fold improvement in MIC compared to the peptide alone [114]. These studies highlight the potential of cleavable linkers in AMP-antibiotic conjugates, enabling controlled drug release and enhanced antibacterial activity, particularly against drug-resistant bacterial strains. These next-generation PDCs hold immense potential for addressing antibiotic resistance, particularly in hospital-acquired infections, biofilm-associated infections, and multidrug-resistant bacterial strains.

Yamauchi et al. [115] designed a membrane-disrupting magainin analog, 9P2-2 (GIKKWLHSPKKFPKKFVKKIMNS-NH2), conjugated to ampicillin via a disulfide bond. By optimizing the antimicrobial peptide type, conjugation position, and linker chemistry, the researchers demonstrated that Amp-SS-9P2-2 exhibited significantly enhanced antimicrobial activity against ampicillin-resistant Acinetobacter baumannii while maintaining low cytotoxicity toward HEK293 cells. The study highlights that selecting non-toxic, membrane-disruptive peptides in combination with a cleavable linker represents a generalizable strategy for developing potent antibiotic agents against emerging infectious diseases. Additionally, the cost-effectiveness of conjugation makes this approach more practical compared to full peptide synthesis. Since the disulfide bond remains stable in the extracellular environment, using enantiomeric peptides could further prevent degradation by proteases. This innovative conjugation method holds potential for reviving abandoned antibiotic candidates that were previously dismissed due to poor outer membrane permeability [116]. Conjugating antibiotics with AMPs is a promising strategy to enhance AMP effectiveness, often resulting in synergistic rather than merely additive effects. For instance, Azuma et al. linked 9P2-2 and oncocin to ampicillin via a disulfide bond, demonstrating significantly improved antibacterial activity against Gram-negative bacteria—reducing the MIC from 10 μM (for the combination) to 2.5 μM with the conjugate. Additionally, antibiotic–AMP conjugates show potential in targeting resilient biofilms [115].

**Figure 3:**
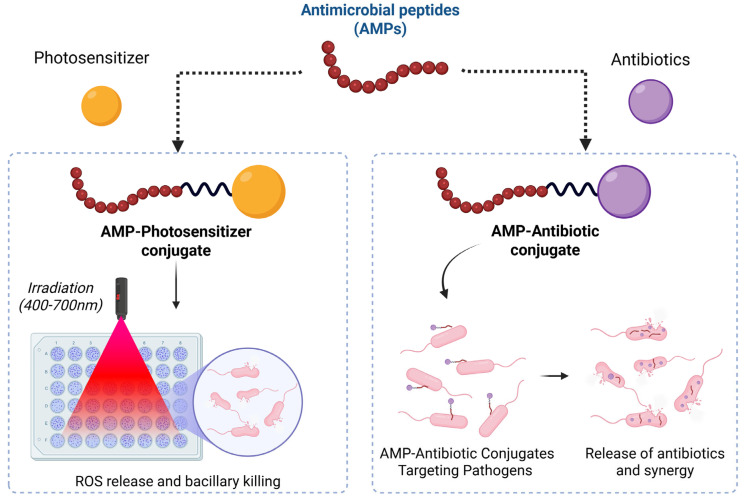
Schematic of antimicrobial peptide (AMP)–antibiotic and AMP–photosensitizer conjugates using cleavable linkers. Cleavage after uptake enhances synergistic killing. AMP–photosensitizer conjugates enable targeted pathogen destruction via ROS generation in photodynamic antimicrobial chemotherapy (PACT). (Created with Biorender.com).

Etayash et al. reported that conjugating the host defense peptide IDR1018 with vancomycin led to more efficient biofilm eradication compared to combination therapy alone. Moreover, these conjugates also promoted immunomodulatory responses, inducing cytokine and chemokine release to bolster host defenses [117]. In recent years, photodynamic therapy (PDT) has been adapted to combat microbial infections through photodynamic antimicrobial chemotherapy (PACT) or photodynamic inactivation (PDI) [118]. Because photosensitizers lack inherent specificity toward particular biological targets, they can be conjugated with AMPs or CPPs that selectively target bacterial or fungal cells. Unlike conventional antibiotics, photosensitizers are unlikely to induce resistance due to the broad-spectrum action and extremely short lifespan (approximately 3 μs) of the reactive oxygen species (ROS) they generate (Figure 3) [119]. Additionally, the effectiveness of photosensitizers under light exposure is influenced by their charge; cationic photosensitizers typically perform better than neutral or negatively charged ones. This property is particularly beneficial when combined with AMPs, as the positive charge of the photosensitizer enhances the conjugate’s affinity for negatively charged bacterial membranes, leading to greater antimicrobial efficacy [120].

### 4.3. Antiviral Peptide–Drug Conjugates

Antiviral PDCs remain underrepresented in drug development pipelines, much like antiviral peptides (AVPs). In most existing cases, the strategy involves chemically linking a known small-molecule antiviral drug to a CPP to facilitate targeted intracellular delivery. PDCs have gained attention as promising antiviral agents due to their ability to enhance drug bioavailability, improve delivery efficiency, and bypass viral resistance mechanisms. These conjugates integrate AVPs with antiviral small molecules, utilizing cleavable or stable linkers to ensure precise and controlled drug release at intracellular targets [121]. Liang et al. engineered indole-conjugated CPPs using click chemistry, significantly enhancing HIV-1 fusion inhibition compared to currently available clinical inhibitors. Their approach involved conjugating small-molecule gp41 inhibitors to the N-terminus of truncated C-peptides, forming a novel conjugated structure. Among these, the 26-residue peptide Indole-T26 demonstrated potent HIV-1 Env-mediated cell–cell fusion inhibition and viral replication suppression at low nanomolar concentrations, matching the potency of T20 (enfuvirtide), the only FDA-approved HIV fusion inhibitor. This study introduces a short peptide-based strategy for HIV-1 fusion inhibition, which may be applicable to developing therapeutics targeting other class I viral fusion proteins [122]. Similarly, Wang et al. designed HIV-1 fusion inhibitors by conjugating small-molecule inhibitors to CPPs, achieving enhanced cellular uptake and antiviral potency. They engineered exceptionally potent small molecule–peptide conjugates demonstrating robust anti-HIV fusion activity in the low nanomolar range. All four conjugates displayed pronounced inhibitory efficacy against HIV-1-induced cell–cell fusion, viral replication, and the formation of the six-helix bundle (6HB) structure. The small-molecule moiety effectively substituted the pocket-binding domain (PBD) of the C34 peptide, maintaining strong anti-HIV-1 fusion activity. In contrast to T20, these hybrid constructs demonstrated greater resistance to proteinase K degradation and maintained potent activity against both T20-sensitive and T20-resistant strains of HIV-1. This strategic integration of small-molecule and peptide-based fusion inhibitors offers valuable insights for the development of next-generation HIV-1 fusion inhibitors targeting the gp41 protein [123].

**Figure 4:**
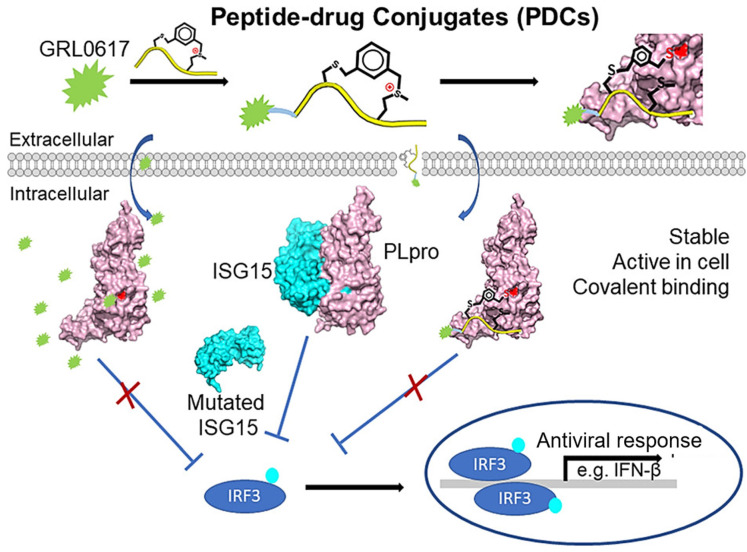
Schematic illustration of PDCs designed to covalently target SARS-CoV-2 PLpro, featuring the inhibitor GRL0617 conjugated to sulfonium-linked peptides based on the PLpro-specific substrate sequence LRGG. Image reproduced with permission from Liu et al. [124].

Liu et al. targeted SARS-CoV-2 papain-like cysteine protease (PLpro) due to its role in viral maturation and immune evasion. They designed a PDC by linking GRL0617 to a sulfonium-tethered peptide derived from the PLpro substrate LRGG. The EM-C and EC-M PDCs exhibited potent in vitro inhibition (IC_50_: 7.40 ± 0.37 μM and 8.63 ± 0.55 μM, respectively), with EC-M covalently binding the PLpro active site (C111) and displaying anti-ISGylation activity. This study introduces PDCs as a novel antiviral strategy, combining peptide specificity with small-molecule potency for effective PLpro inhibition (Figure 4) [124]. Liotard et al. [125] synthesized ester and phosphoramidate peptide conjugates of zidovudine (AZT) and AZT-monophosphate (AZT-MP) for targeted anti-HIV delivery. Ester-based conjugates showed antiviral activity in TK+ cells, correlating with hydrolysis rates, but were not substrates for HIV-protease (PR), acting as prodrugs of AZT. Phosphoramidate conjugates rapidly degraded in buffers, releasing AZT, but exhibited low intracellular drug release and antiviral activity. HPLC confirmed no HIV-PR-mediated cleavage, highlighting the need for improved linker strategies to enhance intracellular AZT-MP delivery [125].

### 4.4. Neurological Disorders

In developing a novel drug delivery system to enable molecules to enter the central nervous system (CNS) via the blood–brain barrier (BBB), it remains challenging to deliver large molecules such as peptides, monoclonal antibodies, and gene therapies across the barrier. Nowadays, constructing BBB-penetrating peptides and shuttle-drug constructs offers promising non-invasive strategies to cross the molecules in the CNS [126]. Chapeau D. et al. [127] recently developed the targeted small-molecule drug conjugate (SMDC), eSOMA-DM1, for treating neuroendocrine tumors. This conjugate consists of an octreotide peptide, which explicitly targets somatostatin receptor subtype-2, linked to the cytotoxic drug DM1 through a chelate linker. The chelate, N3-Py-DOTAGA, minimizes steric hindrance, ensuring efficient receptor binding, and allows for radiolabeling with gallium-68 for diagnostic bioimaging, though radiolabeling with indium-111 showed higher yield and stability. While eSOMA-DM1 demonstrated comparable cytotoxicity to free DM1 at higher doses, its efficacy decreased at lower doses. Additionally, the conjugate showed poor serum stability and altered pharmacokinetics and raised concerns about rapid disulfide bond cleavage, leading to increased systemic toxicity, which needs further attention [127]. In another study, Mendonca et al. fabricated the peptide–porphyrin conjugates (PPC) for enhanced HIV treatment and brain delivery. PPC improves the aqueous solubility of porphyrins, reduces aggregation, and increases cellular uptake. The solid-phase synthesis method using resins was optimized for conjugation, and the DIC/Oxyma activation method yielded higher purity and yields. PPC demonstrated significant BBB translocation without disrupting monolayer integrity. The optimized PPCs had IC50 values ranging from 16–34 µM, linked to membrane affinity and internalization. Out of 14 PPCs, 9 showed no hemolytic activity [128]. Liang Y. et al. [129] developed a VPALR (Val-Pro-Ala-Leu-Arg)–sulpiride conjugate that enhances the drug’s delivery across the BBB and increases its concentration in the brain. Pharmacokinetic studies showed a three-fold increase in bioavailability and prolonged half-life of the drug. The conjugate also exhibited sustained drug release through ester bond cleavage under physiological conditions. It reduced proinflammatory cytokines IL-1β and TNF-α, decreased caspase-3 activity, and mitigated neural apoptosis and inflammation. In depressive mice, the conjugate improved behavioral outcomes compared to sulpiride alone and selectively targeted dopamine D2 receptors in the CNS without peripheral antagonism [129]. Brain metastases occur in half of chronic cancer cases. To address this concern, Zheng M. et al. [130] reported a study validating a peptide conjugated with PTX as a potential treatment strategy for crossing the BBB and blood–tumor barrier. Both in vitro and in vivo models demonstrated that the PDC has a superior ability to cross the BBB compared to the free drug, utilizing LRP1 receptors to facilitate peptide transcytosis. In a mouse xenograft model of lung cancer brain metastases, PDC administration significantly reduced brain tumor proliferation, as evidenced by bioimaging. Histological assessment showed no observable damage to major organs, highlighting the safety of the conjugate for systemic administration [130].

### 4.5. Inflammatory Diseases

PDCs represent a promising class of therapeutics for managing inflammatory diseases, as bioactive peptides can be combined with potent molecules to target inflammation and modulate immune responses with high biocompatibility precisely. Bi J. et al. explored the anti-inflammatory potential of S-allyl-L-cysteine and its garlic acid conjugates, focusing on their mechanism of action and structure-activity relationship. Among 40 synthesized compounds, SMU-8c emerged as the most effective anti-inflammatory agent, potentially inhibiting macrophage nitric oxide production. It explicitly targets TLR2 signaling without affecting TLR3/4 pathways. Enhancing hydrophobicity, extending the carbon chain length, and introducing methoxy groups improved anti-inflammatory activity. Substituents like 1-pentenyl increased activity, while less flexible structures reduced efficacy. Trimethoxy substitution at the third, fourth, and fifth positions was crucial for enhanced activity [131]. Shokri et al. explored the potential of PDCs for targeted anticancer therapy using nonsteroidal anti-inflammatory drugs (NSAIDs) conjugated with linear RGD and Asn-Gly-Arg (NGR) peptides. RGD targets αv-integrin, while NGR targets aminopeptidase N (APN/CD13). Naproxen-spacer–NGR conjugates bind to APN/CD13 through interactions like hydrogen bonding and π–π stacking, with reduced steric clashes. NGR conjugates with spacers exhibited improved activity, specifically against SKOV-3 (CD13+) and HT-1080 cancer cells, highlighting the importance of reducing steric hindrance for effective receptor binding. Although RGD conjugates showed limited efficacy, further investigation into immunogenicity and serum stability is needed to optimize targeting [132]. Rakesh et al. studied quinazolinone-conjugated peptides for their dual potential as antioxidants and anti-inflammatory agents. They modified the peptide template by substituting the second amino acid with residues of varying hydrophobicity, charge, and polarity. The results showed that hydrophobic residues, particularly tryptophan, exhibited high antioxidant potential, while charged (e.g., aspartic acid) and polar (e.g., threonine) residues enhanced anti-inflammatory activity. Anti-inflammatory activity was further increased by longer alkyl chains in the quinazolinone nucleus and blocked C-terminal groups. The study also suggested that antioxidant activity is favored by a polar -COOH group at the peptide’s end, while anti-inflammatory activity prefers a non-polar terminus [133].

### 4.6. Theranostic Peptide Drug Conjugate (TPDC)

TPDCs integrate both therapeutic and diagnostic functions into a single molecule, where a peptide is linked to a drug and a diagnostic agent, enabling targeted drug delivery and real-time monitoring of treatment effects [134,135]. Mitra et al. demonstrated the theranostic potential of ubiquitin conjugated with 2-acetyl-phenyl-boronic acid for combating antibiotic-resistant strains of *Staphylococcus aureus*. The conjugation endowed ubiquitin with bactericidal properties, effectively eradicating both planktonic and small-colony variants of the bacteria. The minimum inhibitory and minimum bactericidal concentrations of ubiquitin were reduced 16-fold upon conjugation. The conjugate was radiolabelled with Gallium-68 to enhance diagnostic capabilities using a NODAGA linker. The radiolabeled conjugate showed high radiochemical purity, stability, and increased uptake compared to its unconjugated form (Table 1) [136]. Pham et al. [137] developed a novel class of theranostic radiopharmaceuticals targeting prostate-specific membrane antigen (PSMA), utilizing the radionuclides 99mTc and 188Re for imaging and radiotherapy applications. These radiotracers demonstrated specific uptake in PSMA-expressing prostate cancer cells with negligible nonspecific binding. The study reported enhanced tumor uptake of the peptide conjugates, accompanied by rapid renal clearance. The diphosphine–dipeptide conjugates (DP1-PSMAt and DP2-PSMAt) offer a significant advantage due to their cost-effectiveness, requiring only bench-top generators and essential kit-based preparation. While DP1 and DP2 exhibited comparable uptake, DP2 accumulated at the renal site. The authors propose that DP1 radiotracers represent superior clinical candidates owing to their reduced off-target effects [137]. Qin Y. et al. developed a ruthenium(II) complex, Ru1-LHRH, conjugated with an LHRH peptide for targeted theranostic applications. This conjugate selectively binds to LHRH-overexpressing A2780 ovarian cancer cells, demonstrating an eight-fold increase in cytotoxicity compared to the parent compound sparing non-target cells. The complex induces apoptosis, facilitates tumor imaging, and is believed to localize in the mitochondria of A2780 cells, where it generates reactive oxygen species and activates Caspase 3/7, key apoptotic markers [138]. Khan et al. developed an injectable theranostic formulation for breast cancer using polyethyleneimine-coated upconversion nanoparticles (UCNP) conjugated with the anticancer drug DOX and electrospun with an EGFR-targeting peptide. The UCNP, sized at 26.75 ± 1.54 nm, demonstrated a photothermal conversion efficiency of 68.8%, generating significant heat (~62.7 °C in 5 min) under 980 nm irradiation. The system exhibited high drug loading (54.56%) and encapsulation efficiency (98.74%) with pH-responsive drug release under acidic conditions. It showed excellent biocompatibility and anticancer solid effects in breast cancer cell lines, and is believed to generate reactive oxygen species to promote apoptosis. Combining photothermal therapy, this targeted formulation offers enhanced cancer treatment with minimized toxicity [139].

**Table 1:** List of different peptide–drug conjugates (PDCs), their respective targeting peptides, therapeutic payloads, and associated disease indications.

Targeting Peptide	Payload	Therapeutic Target	Indication	**Reference**
Heptapeptide (P7)	Docetaxel	Hsp98	Non-Small Cell Lung Cancer (NSCLC)	[75]
RGD	Paclitaxel	αv integrin receptors	Metastatic breast cancer	[76]
Cyclic peptide 3207-86	Camptothecin (CPT)	SSTR2	Anticancer effect	[79]
Octreotide	Doxorubicin	SSTR2	Breast cancer	[83]
[D-Phe6, β-Ala11, Sta13, Nle14] BBN (6–14)	Daunorubicin	Gastrin-Releasing Peptide Receptor (GRP-R)	Prostate and breast cancer	[90]
PD-L1-targeting peptide (PPA1)	Doxorubicin	PD-L1	Colon Cancer	[96]
Oligomeric peptides (KGFRWR)	Doxorubicin	MMP-2 and MMP-9.	Hepatocellular carcinoma (HCC)	[106]
Pep-4	Levofloxacin	Membrane Disruption	Antibacterial	[107]
P14LRR (Fl-PRPRPL-4)	Kanamycin	-	Antibacterial	[112]
T20 peptide (enfuvirtide)	Sapogenin	gp41-specific	Antiviral	[123]
Sulfonium-tethered peptide	GRL0617	Papain-like cysteine protease (PLpro)	Antiviral	[124]
Cell-penetrating peptides (CPPs)	Porphyrins	-	HIV-associated neurocognitive disorders (HAND)	[128]
Arg-Gly-Asx (RGD) and Asn-Gly-Arg (NGR)	Naproxen	Aminopeptidase N	Cancer therapy	[132]
UBI (29–41)	Gallium-68 (68Ga)	-	Theranostic	[136]

## 5. PDC-Based Formulations

The PDC system, composed of a peptide, linker, and drug, functions as a self-delivery mechanism with high drug-loading efficiency, enhancing drug bioactivity while eliminating the need for additional carrier materials. Through self-assembly, PDCs can generate various one-dimensional nanostructures, including micelles, nanofibers, nanotubes, and nanowires. However, systemic administration poses challenges such as inadequate drug accumulation, drug inactivation, and off-target effects, which significantly hinder treatment efficacy and may lead to adverse side effects. In contrast, localized delivery systems offer a promising solution by concentrating the drug at the intended site while minimizing exposure to healthy tissues, thereby improving therapeutic outcomes [140].

**Figure 5:**
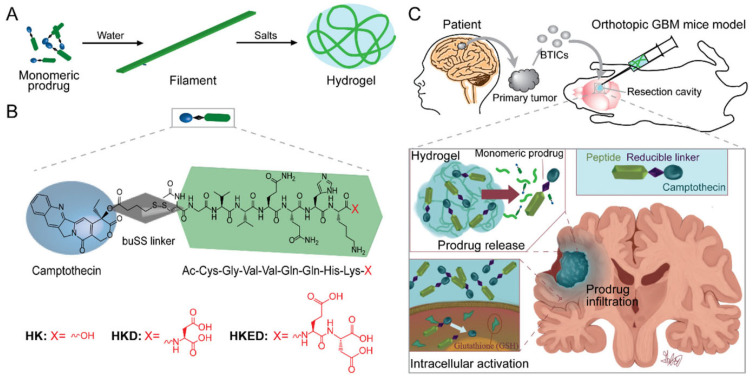
Self-assembling peptide–drug conjugate hydrogels are designed for localized brain tumor treatment. (**A**) Schematic representation of the self-assembly process wherein monomeric prodrugs organizes into supramolecular filaments, subsequently forming a hydrogel upon the introduction of counterions. (**B**) Chemical structures of camptothecin (CPT)-peptide conjugate. (**C**) Schematic depiction of the development of an orthotopic glioblastoma (GBM) mouse model, alongside the proposed mechanism by which the CPT-conjugate hydrogel suppresses tumor recurrence following surgical resection. Image reproduced with permission from Schiapparelli et al. [141].

Self-assembling peptides play a critical role in PDC-based formulations by facilitating hydrogel formation through hydrogen bonding, electrostatic interactions, hydrophobic forces, and π–π stacking. These interactions enable PDCs to form nanofibers, nanotubes, and supramolecular hydrogel networks, creating an effective drug delivery system. A CPT-based self-assembling PDC hydrogel system was developed using a GV2Q2HKD peptide. This system, upon contact with an aqueous solution, formed nanofilaments that entangled into a hydrogel. The hydrogel provided a prolonged drug release effect, suppressing tumor recurrence in glioblastoma multiforme (GBM) models (Figure 5) [141]. Another example is a dexamethasone (Dex) amphiphilic prodrug hydrogel formulated by conjugating Dex with a peptide amphiphile (PA-Dex). This hydrogel exhibited enhanced anti-inflammatory activity with prolonged drug release, reducing systemic immunosuppressive effects [142].

Targeting peptides improve the precision of drug delivery by binding to specific cellular receptors, enhancing drug accumulation at the diseased site. A supramolecular hydrogel bearing the iRGD peptide was designed for tumor penetration. The hydrogel system contained PTX-bearing peptide conjugates that self-assembled into a hydrogel upon exposure to phosphate-buffered saline (PBS). This strategy significantly increased drug retention and cytotoxicity in U87 cancer cells [143]. Another example is a hydrogel system incorporating CPT and DOX for combination chemotherapy. The hydrogel scaffold sustained drug release, enhancing tumor penetration and retention, leading to effective tumor suppression [104]. Bioactive peptides, which possess intrinsic therapeutic effects, have been integrated into PDC-based hydrogels to enhance therapeutic outcomes. A self-adjuvanted peptide vaccine hydrogel (KKEF-TRP2) was formulated to modulate adaptive immune responses against melanoma. The vaccine hydrogel activated dendritic cells and elicited a robust CD8+ T-cell response, effectively inhibiting tumor growth [144]. Another hydrogel system containing YSAYPDSVPMMS peptides targeted the EphA2 receptor on cancer cells, inducing receptor aggregation and activating anti-tumor signaling pathways [145]. The incorporation of bioactive peptides into PDC-based hydrogels follows different strategies, including covalent conjugation, self-assembly, and stimulus-responsive release. Covalent conjugation involves the direct attachment of bioactive peptides to drug molecules through cleavable linkers for controlled release. Self-assembly enables hydrogel formation through non-covalent interactions, improving drug stability and bioactivity. Stimulus-responsive release hydrogels respond to enzymatic, pH, or ionic triggers, allowing for site-specific drug release [140].

## 6. Challenges in Development of PDCs

Several challenges hinder the widespread clinical application and therapeutic efficacy of PDCs. One primary concern is their safety, particularly their biocompatibility. Peptides, derived from amino acids and typically biodegradable in water-soluble form, generally do not exhibit adverse effects. However, when conjugated with drug molecules, there are concerns about whether the biocompatibility and biodegradability of the peptides are retained. Therefore, developing innovative strategies to ensure the biocompatibility and biodegradability of peptides in PDCs is essential. Another challenge is the stability of PDCs, particularly the short half-life of peptides, which limits the distribution and circulation time of PDCs in vivo, offering only a narrow window for drug molecules to enter cells before the peptides undergo enzymatic degradation. Several techniques, such as cyclization (head-to-tail), substitution of unnatural amino acids, disulfide-linked cyclization, mimetic peptides, stapled peptides, and bicycle approaches, have been developed to enhance the half-life of peptides. However, these molecules are small and are often cleared quickly by the kidneys. To address this, combining a prodrug approach with wall materials for novel delivery systems can improve stability and maintain an appropriate size for adequate circulation [46]. Moreover, the efficacy of PDCs must be carefully considered. The linkers in PDCs require specific environmental conditions such as temperature, pH, or enzymes to cleave and release the drug molecule from the conjugate. In some cases, the drug molecule may not be released effectively. Additionally, the rate and efficiency of drug release in target cells are difficult to determine, highlighting the need for appropriate methods to assess the efficacy of PDCs. At present, the oral administration of PDC drugs in protein-based therapies is impeded by enzymatic degradation mediated by gastrointestinal proteases. They are limited to invasive injection methods, which restricts their clinical applicability. To overcome this limitation, designing delivery systems that incorporate suitable carriers or structural modifications is essential to protect PDC drugs from gastrointestinal enzyme degradation and facilitate their absorption into the bloodstream [146]. Ultimately, developing efficient PDCs depends on the design of multifunctional peptides. With advancements in peptide screening and synthesis technologies, intelligent nanomedicine delivery strategies based on PDCs are expected to be widely applied in clinical settings in the future.

## 7. Clinical Status of Peptide–Drug Conjugates

PDCs hold significant potential but are still underdeveloped, with only two approved globally. Lutathera ([^177^Lu] Lu-DOTA-TATE), approved in 2018, targets somatostatin receptor-positive tumors using Peptide Receptor Radionuclide Therapy (PRRT), delivering radiation to damaged tumor cells. The second, Pepaxto (Melflufen), was approved in 2020 for multiple myeloma but was withdrawn in 2021 due to unfavorable results in phase III trials. Similarly, Zoptralin failed to demonstrate an improved safety and efficacy profile compared to DOX alone in phase III trials (NCT01767155). Despite these setbacks, ongoing clinical trials are addressing the shortcomings of PDCs through improved design strategies. For instance, ANG1005 uses Angiopep-2 to cross the BBB and release PTX for treating breast cancer brain metastases, showing improved survival outcomes in patients [50]. Two PDCs currently in clinical development target Sortilin1 (SORT1) receptors: TH1902 and TH1904. The SORT1 receptor is highly expressed in several cancers, including breast and ovarian cancer. TH1902, which carries DTX as its cytotoxic payload, has received fast-track designation from the FDA for treating SORT1-positive patients with advanced solid tumors that are resistant to standard therapies. This PDC is currently being evaluated in phase I clinical trials. Meanwhile, TH1904, which delivers DOX, is still in the preclinical research phase. Additionally, other PDCs under clinical investigation include synthetic analogs of natural peptide ligands conjugated to chemotherapeutic agents such as DOX and PTX. However, the clinical outcomes of these PDCs have been inconsistent, indicating that challenges remain in translating their promising pharmacodynamic properties into significant therapeutic benefits for patients [36]. As better re-engineering approaches emerge, many PDC candidates continue to advance in clinical development, as listed in Table 2.

## 8. Future Perspectives

The trajectory of PDC research heralds a transformative era, underpinned by advancements in molecular design, nanotechnology, and computational biology. A significant focus lies in the incorporation of humanized antibodies into PDC frameworks, circumventing the immunogenic pitfalls of murine analogs while amplifying therapeutic precision and safety profiles. This humanization not only mitigates immunological concerns but also prolongs systemic circulation, thereby enhancing drug bioavailability. Emerging peptide scaffolds such as Affibodies and ADAPTs offer high-affinity tumor targeting, precise drug loading, and ease of production, making them promising for next-generation PDCs [147]. In parallel, strategies to augment biological stability, integrating robust linkers and protective nanodrug delivery systems, address the inherent vulnerability of PDCs to enzymatic degradation. These multifunctional nanosystems, designed for targeted and controlled release, have the potential to synergize PDCs’ specificity with enhanced pharmacokinetic attributes, revolutionizing therapeutic outcomes in oncology and beyond. The integration of computational methodologies, particularly artificial intelligence (AI), marks a pivotal frontier in PDC innovation. AI-driven platforms facilitate the rational design of novel peptides, payloads, and linkers, optimizing interactions between ligands and receptors while predicting pharmacodynamic behaviors with unparalleled precision. Concurrently, the evolution of bispecific and dual-drug-loaded PDCs underscores the field’s shift toward combating therapeutic resistance. By engaging multiple receptors or employing diverse cytotoxic payloads, these constructs amplify cellular internalization and dismantle cancer resistance mechanisms. Moreover, the advent of multifunctional linkers, capable of drug tracking or catalyzing specific biological reactions, embodies the push for theranostic capabilities within PDCs. Collectively, these advancements foreshadow a paradigm wherein PDCs are not only potent therapeutic agents but also diagnostic tools, embodying the confluence of targeted precision, systemic stability, and therapeutic adaptability.

## 9. Conclusions

PDCs epitomize a modern therapeutic paradigm, seamlessly integrating the molecular precision of peptides with the pharmacodynamic potency of small molecules to address pathologies of formidable complexity, including oncological and neurodegenerative disorders. Their small size and unparalleled modularity confer an adaptive advantage, enabling the surmounting of limitations intrinsic to singular therapeutic modalities. Notwithstanding their transformative potential, PDCs remain encumbered by challenges such as immunogenicity, off-target toxicity, and bioavailability constraints. Resolving these impediments necessitates sophisticated advancements in peptide scaffolding, linker stability, payload specificity, and formulation strategies. Moreover, the strategic combination of PDCs with immunomodulatory and genomic interventions augurs unprecedented therapeutic synergies, heralding a paradigm shift in the treatment of recalcitrant diseases.

## Figures and Tables

**Table 2:** List of various peptide–drug conjugates currently in clinical trials and those approved for therapeutic use.

Clinical Trials Gov. ID	Peptide–Drug Conjugate	Application	Phase	Drug Component	Sponsors
NCT02048059	ANG1005	Targets breast cancer cells with relapsing brain metastases	II	Paclitaxel	Angiochem Inc
NCT03613181		For newly diagnosed leptomeningeal carcinomatosis with prior brain metastases	III		
NCT04706962	TH1902	Therapy for solid tumors or cancers expressing the SORT1 receptor	I	Docetaxel	Theratechnologies
NCT05465590	MB1707	Targets the SDF-1/CXCR4 pathway to inhibit tumor growth and metastasis	I	Paclitaxel	Mainline Biosciences, Inc.
NCT05725070	212Pb-NG001	Theranostic salvage therapy for metastatic castration-resistant prostate cancer using PSMA-targeted 212Pb-NG001	0/I	212Pb-NG001	ARTBIO Inc.
NCT01480583	GRN1005	Potential monotherapy or combination with trastuzumab for breast cancer brain metastases	II	Paclitaxel	Angiochem Inc
NCT01497665		Patients with non-small cell lung cancer and brain metastases.	II		
NCT05518071	FLUOPANC	Fluorescent marker for bile duct and pancreatic tumor surgery	I	Fluorophore ZW800-1	Leiden University Medical Center
NCT01698281	AEZS-108 (Zoptarelin DOX)	Chemotherapy for triple-negative breast cancer	II	DOX	AEterna Zentaris
NCT01767155		Second-line treatment for endometrial cancer	III		
NCT03486730	BT1718	Treatment of advanced solid tumors	I/IIa	DM1	Cancer Research UK
NCT00710125	GPX-150	Curing solid tumors	I	Modified analog of DOX	Gem Pharmaceuticals
NCT02267083		Therapy of soft tissue sarcoma	II		
NCT06326190	^177^Lu-DOTATATE	Recurrent Meningioma	II	^177^Lu	European Organization for Research and Treatment of Cancer—EORTC
NCT04529044		Treating recurrent or stage 4 breast cancer	II		OHSU Knight Cancer Institute
NCT02489604		Curing advanced gastroenteropancreatic neuroendocrine tumors	II		Istituto Scientifico Romagnolo per lo Studio e la cura dei Tumori
NCT04385992		Post-surgical treatment for resectable pancreatic neuroendocrine tumors	II		IRCCS San Raffaele
NCT06460467		Dosimetric calculation for treating neuroendocrine tumors or meningiomas	I		Central Hospital, Nancy, France
NCT02736500		Treating aggressive gastroenteropancreatic neuroendocrine tumors	I-II		Istituto Scientifico Romagnolo per lo Studio e la cura dei Tumori
NCT04544098		Gastroenteropancreatic, bronchial, or unknown primary neuroendocrine tumors metastasized to the liver	I		Memorial Sloan Kettering Cancer Center
NCT04180371	BT5528	Treating advanced solid tumors exhibiting EphA2 expression	I/II	Monomethyl auristatin E	BicycleTx Limited
NCT04552847	[^18^F]AlF-NOTA-octreotide	PET imaging of neuroendocrine tumors	II/III	^18^F	Universitaire Ziekenhuizen KU Leuven
NCT00918281	[^18^F]Fluciclatide	Solid tumor PET imaging	II	^18^F	GE Healthcare
NCT01633255		Imaging kidney cancer	I/II		National Cancer Institute
NCT01633255	[^18^F]RGD-K5	PET imaging	II	^18^F	Siemens Molecular Imaging
NCT02381236	G-202 (mipsagargin)	Non-invasive multiparametric prostate magnetic resonance imaging (mpMRI)	II	Thapsigargin	GenSpera, Inc.
NCT03445884	^68^Ga-NODAGA-E[cyclo(RGDyK)]	PET imaging	II	^68^Ga	Rigshospitalet, Denmark
NCT02749019	^68^Ga-NOTA-BBN-RGD	PET imaging	I	^68^Ga	Peking Union Medical College Hospital
NCT02936323	PEN-221	For somatostatin receptor 2 expressing higher stages of cancers, including neuroendocrine and small cell lung cancers	I/IIa	DM-1	Tarveda Therapeutics
NCT03273712	^90^Y-DOTATOC	Radionuclide therapy for patients with somatostatin receptor-positive tumors	II	^90^Y	University of Iowa
NCT04740398	CBP-1008	Later stages of solid tumors	I	MMAE	Coherent Biopharma (Suzhou) Co., Ltd.
NCT04928612	CBP-1018	Later stages of solid tumors	I	MMAE	Coherent Biopharma (Suzhou) Co., Ltd.
NCT03784677	SOR-C13	Later stages of malignant solid neoplasm	I	MMAE	M.D. Anderson Cancer Center
NCT05079698	^177^Lu-PSMA-617	Prostate cancer	I	DOTA	Memorial Sloan Kettering Cancer Center
NCT02742168	^99m^Tc-3PRGD2	Breast cancer	I	^99m^Tc	First Affiliated Hospital of Fujian Medical University

## Data Availability

Not applicable.
